# Toxicity Assessment of Cadinene Sesquiterpenes from *Eupatorium adenophorum* in Mice

**DOI:** 10.1007/s13659-014-0050-2

**Published:** 2014-12-12

**Authors:** Can-Bin Ouyang, Xiao-Man Liu, Qi Liu, Jie Bai, Hou-Yong Li, Yuan Li, Qiu-Xia Wang, Dong-Dong Yan, Lian-Gang Mao, Aocheng Cao, Mei-Xia Guo

**Affiliations:** 1 Department of Pesticides, Institute of Plant Protection, Chinese Academy of Agricultural Sciences, Ministry of Agriculture, and State Key Laboratory for Biology of Plant Diseases and Insect Pests, Beijing, 100193 People’s Republic of China; 2 National Engineering Laboratory for Crop Efficient Water Use and Disaster Mitigation, and Key Laboratory of Dryland Agriculture, Ministry of Agriculture, Institute of Environment and Sustainable Development in Agriculture, Chinese Academy of Agricultural Sciences, Beijing, 100081 People’s Republic of China; 3 Department of Agriculture, Bio-engineering and Chemistry, University of Liege-Gembloux Agro-Bio Tech, 5030 Gembloux, Belgium; 4 Department of Pathology, Shandong Academy of Occupational Health and Occupational Medicine, Jinan, 250062 People’s Republic of China

**Keywords:** *Eupatorium adenophorum*, Sesquiterpenes, Histopathology, Environmental toxicity, Biopesticide

## Abstract

This study evaluated toxic efficacy of *Eupatorium adenophorum* extracts, against the Kunming mice. In acute study, we firstly tested median lethal dose (LD_50_) in mice of three cadinene sesquiterpenes 2-deoxo-2-(acetyloxy)-9-oxoageraphorone (DAOA), 9-oxo-agerophorone (OA) and 9-oxo-10,11-dehydro-agerophorone (ODA) from *Eupatorium adenophorum* (Ea). DAOA (215–4640 mg/kg BW, given orally) showed lowest LD_50_ at 926 mg/kg BW for male mice in contrast with OA (1470 mg/kg BW) and ODA (1470 mg/kg BW). In sub-acute study, repeated doses (75–300 mg/kg BW, for 7 days) of DAOA/OA increased blood parameters, liver and spleen index in dose dependent relationship, along with decrease in thymus index. The blood biochemical and histopathological examination showed that DAOA/OA dose 300 mg/kg BW significantly causes pathological changes of hepatic lobules and hepatocytes, which are consistent with cholestasis and hepatic injury. 75 mg/kg dose of DAOA/OA was found to be approximately/totally safe over the span of 7 days treatment showing no change in all above described parameters. Cadinene sesquiterpenes guarantee low risk to environment as a type of low toxic botanical components, which may find potential application in biopesticides development field.

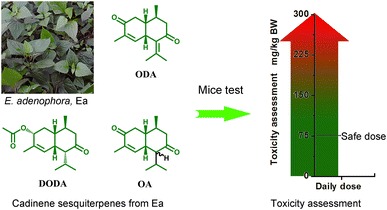

## Introduction

As a composite plant, the fast growth of *Eupatorium adenophorum* (Ea), is changing overall biodiversity and ecological balance in the forests and pastures in southwestern China [1, 2]. It prompts increasing requirements to control Ea. Many control technologies have been developed, such as chemical, physic and biology control, as well as comprehensive utilization [3–5]. Comprehensive utilization is the best control policy, because it does not only consuming or using up invasive weeds but also transferring hazardous weeds into valuable products. Our research group has been pushing hard for Ea industrialization promotion, and made a breakthrough in producing animal feeds, fertilizers, charcoal, chlorogenic acid and dyestuff, etc. in cooperated with several companies. Although extracts of Ea have been reported to possess insecticidal, antibacterial properties and potential anti-inflammatory [6–12], and may have valuable implications for the development of novel biopesticides, no pesticide products have appeared so far. This is because huge investment for drug development and registration limits the progress. On the other hand, identification of bioactive compounds and their environmental risk assessment is still limited.

**Fig. 1:**
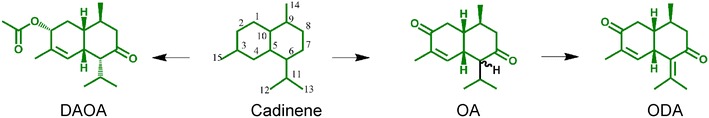
Molecular structures of three cadinene sesquiterpenes in Ea leaves

Indeed, there are more than 20 different sesquiterpenes in Ea, of which most have same molecular skeleton of cadinene (Fig. 1) [13–19]. Among them, 9-oxo-10,11-dehydro-agerophorone (ODA) in Ea is a main poisonous components inducing the diarrhea or even death of livestock [20–24]. It has been identified as the hepatotoxicant to rats very early [25]. However, toxic size and environmental security of these sesquiterpenes have not been reported. Two typical compounds are 2-deoxo-2-(acetyloxy)-9-oxoageraphorone (DAOA) and 9-oxo-agerophorone (OA), respectively. The main differences between DAOA and OA on molecular structure are the 2-acetoxy and 2-carbonyl groups, distinguishing feature between ODA and OA is the presence or absence of an unsaturation 6–11 bond in conjugation with a 7-oxo function. Our previous studies have reported the extraction and separation methods of sesquiterpenes, and demonstrated that DAOA, OA and ODA were mainly distributed in Ea leaves with 0.63–1.99 % of mass percentage in dry leaves [26]. If these sesquiterpenes could find application in biology medical or biopesticides fields, Ea will have greater development value. Herein, we investigate the toxicity efficacy of cadinene sesquiterpenes to mice, to evaluate the environmental security on non-target organism. It indicates that these cadinene sesquiterpenes from Ea are potential candidates for botanical biopesticides.

## Results and Discussion

### Effect of Sesquiterpenes on Acute Toxicity

Three sesquiterpenes were administrated to Kunming mice as mixed with tween-water in acute and sub-acute test. Three compounds showed similar toxic efficacy to mice. The physical activity was significantly reduced and the response was dose dependent resulting in death on higher doses. For 215 and 464 mg/kg BW (body weight) of DAOA, OA and ODA, some animals reduced the intake of diet, peed dark yellow urine, and then rehabilitated to normal state after 2–3 days. As shown in Table 1, there were no animals died of 215 and 464 mg/kg dosage for 14 days. For moderate single dose 1000 mg/kg, animals were icteric, dull, and appeared rough of hair coat and gait ataxia, etc. there were more or less 50 % of the mice died in 2 weeks. As shown in Table 1, there were 8/10, 5/10 and 5/10 mice die of DAOA, OA and ODA, respectively. For large single dose ≥2150 mg/kg, all animals appeared remarkable symptoms of inability to stand, gait ataxia, convulsion and hyperspasmia for the first 15 s. The first animals died after 0.5 h, and the rest died all in next 2 h. The oral LD_50_ (median lethal dose) of DAOA, OA and ODA were determined by Horn’s method [27]. As shown in Table 1, males’ mortality of four DAOA dose levels (215, 464, 1000 and 2150 mg/kg) were 0, 0, 3 and 5 (bold), respectively, thus the LD_50_ is determined to 926 mg/kg on Horn’s table [27]. The females’ mortality of 0, 0, 5 and 5 (bold), corresponding to four levels of 215, 464, 1000 and 2150 mg/kg of DAOA, indicate that the LD_50_ is 681 mg/kg. Similarly, the LD_50_ of OA and ODA are 1470 and 1470 mg/kg for males, 681 and 681 mg/kg for females, respectively (mortality are show in bold). So far, this is the first report of cadinene sesquiterpenes’ LD_50_ on mice. DAOA, OA and ODA extracted from Ea exhibit low toxicity, and DAOA has the highest toxicity. Although molecular mechanism of action is unknown, toxicity is more affected by substituent group on cadinene skeleton, and less affected by degree of unsaturation. For example, the presence of certain hydroxyl groups on the sesquiterpenes ring enhances antioxidant activity [28–30]. Furthermore, as ODA is a hepatotoxicant, DAOA and OA could also probably induce hepatic lesion [31].

**Table 1:** The acute assay results of mice on administration of DAOA, OA and ODA for 14 days

Dose (mg/kg)	Mice amount	DAOA		OA		ODA	
		Male	Female	Male	Female	Male	Female
215	5	**0**	**0**	0	**0**	0	**0**
464	5	**0**	**0**	**0**	**0**	**0**	**0**
1000	5	**3**	**5**	**0**	**5**	**0**	**5**
2150	5	**5**	**5**	**5**	**5**	**5**	**5**
4640	5	5	5	**5**	5	**5**	5
LD_50_ (mg/kg)		926	681	1470	681	1470	681

Mortality of mice in different group, 95 % confidence limit, n = 5

### Effect of Sesquiterpenes on Sub-acute Toxicity

On the third day, animals in DAOA3 and OA3 group began to show toxic symptoms of not shiny hair, reducing diet, weight loss, gait ataxia, huddle and reduction, etc. As time goes on, the symptoms of animals remarkably deteriorated and mice began to die in DAOA3 and OA3 groups at the 4th day. No animals died of DAOA/OA at 75, 150 mg/kg dose, and typical symptoms of jaundice and photosensitization, along with yellowish skin, eyes and paws have lasted for several weeks. At the end of 7th day, there are 65 and 30 % mice died of total 2150 mg/kg dose in DAOA3 and OA3 groups, respectively. In contrast, when 2150 mg/kg dose was given orally in a single dose, 100 % animals died during 2 h. It indicates that the safety dose should not exceed 300 mg/kg BW. Once detoxification system is overburdened, animal dies, e.g. excessive appetite of Ea could cause anorexia, inability to stand and even death of horses [20–24].

The blood routine and biochemical examinations were performed to investigate the cellular or metabolic change as intra-cellular enzymes are very sensitive to some chemicals, especially pesticides [32], and their activities can be detected in a small amounts. The results of blood routine examination of survivals were shown in Table 2. For DAOA dose 300 mg/kg (DAOA3 group), many routine hematological parameters show significant difference versus control group. RBC and HGB show significant decrease (p < 0.01), while CHE and WBC show remarkable increase (p < 0.01). Meanwhile, there was no significant difference in any group of OA. It indicates that the animals had significant inflammatory reactions at higher dose of DAOA [33]. The blood routine test also demonstrated a potential risk on immune system of 300 mg/kg BW DAOA, due to the decreases of LYM, RBC and HGB in serum.

**Table 2:** The results of mice blood routine tests of DAOA and OA

Test item	CK	DAOA1	DAOA2	DAOA3	OA1	OA2	OA3
CHE (×10^3^ U/L)	6.07 ± 0.66	7.15 ± 0.99	7.14 ± 0.74	7.31 ± 1.07**	5.57 ± 0.74	5.79 ± 0.53	6.32 ± 0.65
WBC (×10^9^/L)	6.81 ± 0.95	7.48 ± 1.19	12.44 ± 3.52	16.86 ± 4.80**	7.81 ± 2.79	7.16 ± 1.14	9.73 ± 2.90
LYM (×10^9^/L)	6.66 ± 1.84	6.68 ± 2.29	5.73 ± 2.17	4.42 ± 0.79**	5.41 ± 1.67	5.04 ± 0.71	4.86 ± 1.70
RBC (×10^12^/L)	8.89 ± 0.75	8.81 ± 0.97	7.60 ± 1.16	7.08 ± 1.08**	8.53 ± 1.08	8.90 ± 0.58	8.67 ± 0.48
HGB (g/L)	130.0 ± 4.0	131.8 ± 8.8	119.9 ± 7.6	108.5 ± 13.6**	124.8 ± 14.1	128.8 ± 7.9	126.5 ± 8.6
PLT (×10^12^/L)	1.46 ± 0.36	1.37 ± 0.42	1.19 ± 0.55	1.70 ± 0.28	1.02 ± 0.29	1.43 ± 0.13	1.29 ± 0.38

Each value represents the mean ± S.D. of mice

** Significantly different from CK group (P < 0.01). n = 10

**Fig. 2:**
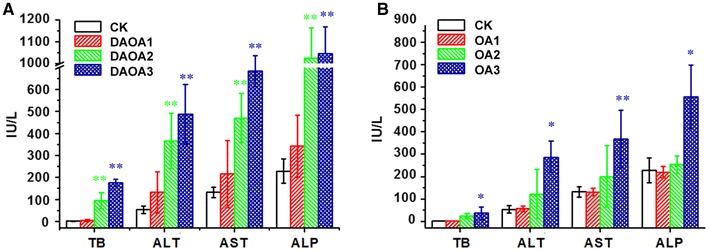
Serum TB, ALT, AST and ALP profiles of mice. **a** DAOA **b** OA. Each value represents the mean ± S.D. of ten mice per group. *Significantly different from CK group (P < 0.05); **Significantly different from CK group (P < 0.01)

The results blood biochemical examinations of survivals were shown in Fig. 2. In sub-acute tests, the biochemical parameters e.g. liver function and bilirubin exhibit obvious dose-dependent relationship on administration of DAOA and OA. DAOA toxic size is larger than OA. DAOA significantly raised TB (p < 0.01), AST (p < 0.01), ALT (p < 0.01) and ALP (p < 0.01) at moderate dose of 150 mg/kg, whereas OA significantly raised TB (p < 0.05), AST (p < 0.01), ALT (p < 0.05) and ALP (p < 0.05) at highest dose of 300 mg/kg BW. Thus, the increase in the TB, AST, ALT and ALP levels were more marked on oral administration of DAOA. As shown in Fig. 2b, 75 mg/kg dose of OA was found to be totally safe over the span of 7 days treatment showing no change in all above described parameters. Rise in TB suggests its damaging effect on hepatobiliary system [34]. Significant change in the activity of ALT, AST and ALP suggests that the DAOA and OA cause cellular damage [35]. The DAOA significantly raised TB, ALT, AST and ALP at a lower dose than OA, suggesting its higher hepatotoxicity [20–24]. The observation provides the biochemical basis of jaundice.

**Fig. 3:**
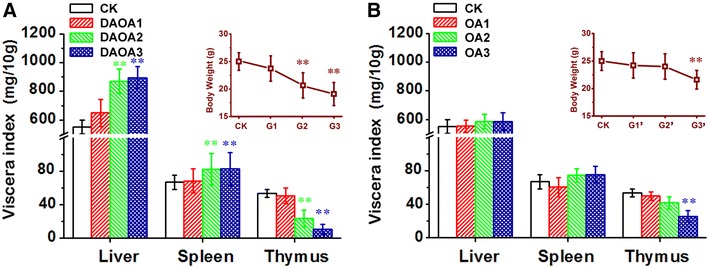
Viscera index of animals in 7 groups after 7 days of drug administration. **a** DAOA **b** OA. The inset is the animal’s body weight of 7 groups. Each value represents the mean ± S.D. of 40 animals. *Significantly different from CK group (P < 0.05); **Significantly different from CK group (P < 0.01)

We observed and handled with statistics the viscera changes of mice. The viscera changes are mainly hepatomegaly, gallbladder enlargement, and severe thymus atrophy at 300 mg/kg dose of DAOA and OA. As shown in Fig. 3, oral administration of DAOA and OA for 7 days continuously in different doses (300, 150, 75 mg/kg BW) showed dose dependent viscera index in mice. As shown in Fig. 3a, all viscera index exhibit significant changes (p < 0.01) on administration of 150 mg/kg BW DAOA. In contrast, only thymus index exhibits significant decrease (p < 0.01) of 300 mg/kg BW OA. In addition, the insets show that drug dose induced significant mice weights decrease (p < 0.01), is 150 and 300 mg/kg for DAOA and OA, respectively. The body weight decrease was probably caused by reduced diet [36]. The toxic dose inducing significant change is 150 and 300 mg/kg for DAOA and OA, respectively, and the trend is in line with biochemical parameters. Thus, it clearly demonstrated that DAOA/OA could also cause hepatic damages. In addition, obvious atrophy of two primary immune organs of thymus and spleen also suggests a potential risk immune system at higher dose of DAOA, which was in line with decreases of LYM, RBC and HGB in serum.

**Fig. 4:**
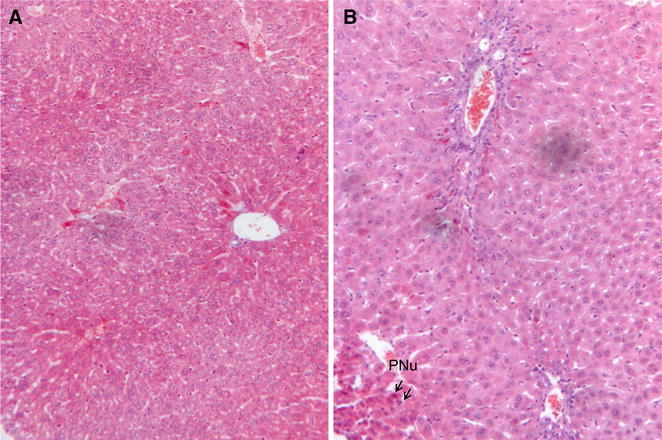
Photomicrograph of HE staining histological sections in livers. **a** control animal, uniform pattern of polyhedral hepatocyte radiating from the central vein towards the periphery; **b** intoxicated animal in DAOA3 group, portal-central vein bridging necrosis, focal necrosis of liver cells and pycnotic nucleus (PNu). Magnification 100×

**Fig. 5:**
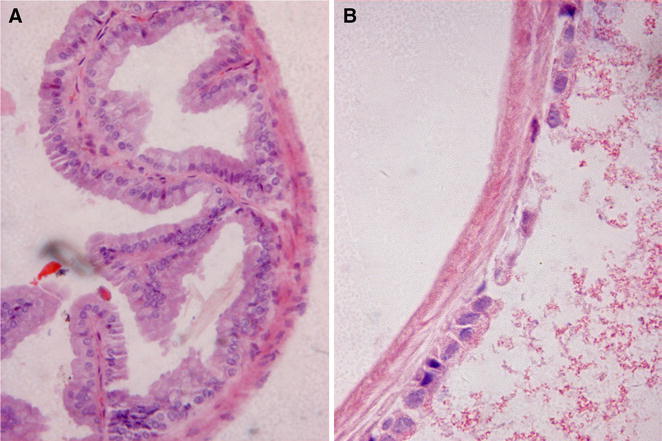
Photomicrograph of HE staining histological sections in gallbladders. **a** control animal, normal gallbladder cells with uniform size and clear mucosa; **b** intoxicated animal in DAOA3 group, typical extension and partial leakage of epithelial cells and cytoplasm. Magnification 100×

As DAOA is more hepatoxic than OA and ODA, the histological findings were undertaken on 300 mg/kg BW administration of DAOA. Histological pictures of liver and gallbladder in CK and DAOA3 groups were presented in Figs. 4 and 5, respectively. As shown in Fig. 4a, normal animals exhibited clear cut hepatic lobules, separated by interlobular septa and traversed by portal veins. Within the lobule, hexagonal array of hepatic plates, radiating towards periphery from a central vein were visible. The hepatocytes were polyhedral, and the nuclei were round with a roughly uniform size. At the dose of 300 mg/kg, alterations of hepatic pathology appear in Fig. 4b, obviously. Figure 4b shows fibrogenesis, vessel dilation, bridging necrosis and focal necrosis of liver cells, obviously. The hepatic cords were disrupted at many areas, with pycnotic nucleus (PNu) in hepatocytes. The gallbladder histological pictures were presented in Fig. 5a. It shows clear figure of gallbladder tissue consisted of the round nuclei with uniform size, clear brush border, mucous cells of the epithelium, apical part of the columnar cells. Figure 5b shows cholecystectasis with thinner mucosa, and typical partial leakage of gallbladder epithelial cells (dilation of bile ducts with degenerative changes in the lining epithelium). The macroscopic observations of histopathology allowed visualized confirmation of organ tissues injury [28–30] on oral administration of 300 mg/kg daily 7 days. It suggests that the DAOA might be interacting primarily with the liver tissue cell’s membrane, and cellular metabolic pathways [32, 37].

The histological changes are consistent with the results of hematological and biochemical examinations. Elevation in plasma TB concentration is due to the onset of periportal necrosis of liver [38, 39]. ALP activities increase in case of hepatic cell damage and the obstruction of bile ducts due to fibrogenesis [40, 41]. Inflammatory cytokines play a key role in fibrosis, given that persistent inflammation almost always precedes fibrosis [42]. Denaturation and necrosis of hepatocyte cause jaundice [20–24]. The observations on elevated bilirubin level, increase in the activities of plasma enzymes and hepatic lesions imply that DAOA/OA/ODA elicited hepatotoxicity associated with cholestasis in mice [43–45]. In experimental animals, the changes of clinic symptoms, viscera index, serum parameters and histological morphologies may differ depending on the exposure dose and time, which are similar to some pesticides [32, 46–48]. In the view of their low toxicity to non-target organism, such as human body, the sesquiterpenes of DAOA, OA and ODA are potential biopesticides or biological insecticides. In addition, cadinene sesquiterpenes have similar toxic effect, so the base of toxicity is probably the cadinene skeleton. The structure-active relationship need be further investigated for the development of novel natural pesticides of lower environmental risk [49].

## Conclusion

In summary, we first detected toxicity of three sesquiterpenes and evaluated environmental safety in acute and sub-acute tests. DAOA and OA from Ea were identified as new toxic hepatotoxicants to mice. LD_50_ of DAOA is 926 mg/kg BW to male mice, and its toxicity is about 1.6 times as much as OA and ODA. In sub-acute tests, hepatotoxicity exhibited an evidently dose-dependent relationship at oral dose 75–300 mg/kg BW, continually for 7 days. At higher dose of 300 mg/kg, DAOA significantly changed routine blood parameters, biochemical parameter and liver index of mice, along with immune organs index of spleen and thymus. It indicated that higher dose 300 mg/kg induce not only hepatotoxicity, but also potential immunity toxicity. Pathology changes at higher dose 300 mg/kg indicated that hepatocytes necrosis, fibrogenesis in hepatic lobules, and cholecystectasis with partial leakage of gallbladder epithelial cells were the basis for hepatic function damage. 75 mg/kg dose of DAOA/OA showed litter/no change in all described parameters, indicating rarely security risks to non-target organisms, such as human body. These studies demonstrated that cadinene sesquiterpenes with insecticidal and antibacterial properties may find potential applications as biopesticides on comprehensive utilization of Ea.

## Experimental Section

### Materials and Methods

Reagent grade chemicals and solvents were purchased from Aldrich, and Acros Chemical Co., Inc. The chemicals were used as received without further purification. Biochemistry Analyzer AU640 and Microscope BX50 were from Olympus Optical Co., Ltd., total bilirubin (TB), aspartate aminotransferase (AST), alanine aminotransferase (ALT) and plasma alkaline phosphatase (ALP) kit were from Nanjing Jiancheng Bioengineering Institute, China. DAOA, OA and ODA were isolated from *E. adenophorum* leaves, and purified (98 % purity) by the HPLC by our group [26]. Male and Female Kunming mice (SPF level), weighing from 20 to 24 g were obtained from Central Pharmacy (Experimental Animal Center of Shandong University, China). Before experiments, animals of ambrosia were housed for 14 h in cages, with sawdust-covered floors. They were maintained in a colony room at 22 ± 2 °C under conditions of controlled humidity (70 ± 5 %). Access to water and standard commercial pellet chow was free. This study was carried out in strict accordance with the recommendations in the Guide for the Care and Use of Laboratory Animals of the National Institutes of Health. The protocol was approved by the Committee on the Ethics of Animal Experiments of Shandong Academy of Occupational Health and Occupational Medicine (Permit Number: SYXK (Lu) 2012 0001). All surgery was performed under sodium pentobarbital anesthesia, and all efforts were made to minimize suffering.

### Experiment Design

The sesquiterpenes were suspended in 0.5 mL drug vehicle (0.5 % aqueous solution of tween-80) with different concentration. The experiments were divided into acute and sub-acute toxicity study. The acute toxicity was studied by Horn’s method, as it was not only useful for calculation but also saving animals [27]. According to Horn’s method, single dose (215, 464, 1000, 2150 and 4640 mg/kg, with a factor of 2.15 between dosage levels) of DAOA/OA/ODA was given to each group (n = 10, five females and five males) of animal after fasted overnight and they were observed for change in diet, general behavior and mortality in 2 weeks. Among four dosage levels in a geometric series (five animals per level), Horn’s logarithms table could determine the LD_50_ with a 95 % confidence limit. In the sub-acute study, mice were divided into seven groups, having 40 mice in each. The drug vehicle was given in experimental control group (CK) and different dose of DAOA/OA was given in drug treated, 6 experimental groups (DAOA1, DAOA2, DAOA3, OA1, OA2, OA3). Animals in DAOA1, DAOA2 and DAOA3 group were single dosing 75, 150 and 300 mg/kg of DAOA daily, respectively. Animals in OA1, OA2 and OA3 group were single dosing 75, 150 and 300 mg/kg of OA daily, respectively. Sub-acute experiments lasted for 7 days, and then the survivals were weighed and dissected for toxicity study. Change in body weight was noted and blood samples were collected from each mouse on 7th day. The survivals in 7 groups were picked eyeball blood, to get hematology and blood biochemistry examination, followed by determination of serum enzyme levels.

### Routine Blood and Biochemical Analysis

Routine blood parameters were assessed including choline esterase (CHE) content, lymphocyte (LYM) count, red blood cell (RBC) count, and white blood cell (WBC) count, hemoglobin content (HGB), and platelet (PLT). Hepatic marker enzymes are primarily used to evaluate hepatic damage. For hepatic function, TB, ALT, AST and ALP were detected. All biochemical parameters were determined using auto analyzer using different methods viz kinetic rate method for ALT and AST, TB by Jendrassik and Grof method.

### Histopathological Analysis

Animals were weighed and then sacrificed by cervical dislocation. The organ of heart, liver, gallbladder, thymus gland, spleen, adrenal gland, etc. were carefully dissected out, washed with isotonic saline, blotted dry, and weighed, respectively. The viscera index (organ weight/10 g of body weight) of each mouse was calculated to explore the target organs and reflect the comprehensive intoxication. Then a small piece of liver or gallbladder was fixed in 10 % neutral buffered formalin for 24 h and rinsed with 70 % ethanol, dehydrated in serial dilutions of ethanol before embedding in paraffin wax. Paraffin blocks of the tissues were sectioned at 4 μm thickness, which were rehydrated in distilled water and stained with Hematoxylin–Eosin (H–E) before served for collect histopathological tissue images under Olympus Microscope BX50.

### Statistical Data Analysis

In each assay, the experimental data represent the means of 40 and 10 independent assays (n = 40, 10) ± standards deviations, in acute and sub-acute tests, respectively. Then these data were analyzed using SPSS 13.0 for Windows (Chicago, IL). The statistical significance has been determined using one-way analysis of variance (ANOVA). Differences were considered significant at the level p < 0.05 and very significant at the level p < 0.01. Means comparison was done using Duncan Test.
